# *Schistosoma haematobium* infection levels determine the effect of praziquantel treatment on anti-schistosome and anti-mite antibodies

**DOI:** 10.1111/j.1365-3024.2012.01363.x

**Published:** 2012-06

**Authors:** N RUJENI, N NAUSCH, N MIDZI, T MDULUZA, D W TAYLOR, F MUTAPI

**Affiliations:** 1 Institute of Immunology and Infection Research, Centre for Infectious Diseases, School of Biological Sciences, University of Edinburgh, Ashworth LaboratoriesEdinburgh, UK; 2 National Institute of Health ResearchHarare, Zimbabwe; 3 Department of Biochemistry, University of ZimbabweHarare, Zimbabwe; 4 Division of Pathway Medicine, School for Biomedical Studies and Edinburgh Infectious Diseases, University of Edinburgh, Ashworth LaboratoriesEdinburgh, UK

**Keywords:** *atopy*, Dermatophagoides pteronyssinus, *IgE*, *IgG4*, *infection*, *praziquantel*, Schistosoma haematobium

## Abstract

*Field studies show an association between schistosome infection and atopy, but the effects of anti-helminthic treatment on this association have not yet been investigated in human populations with different schistosome endemicity levels. This study aimed to compare the effects of anti-helminthic treatment on responses directed against the house dust mite* Dermatophagoides pteronyssinus *(Derp1) and* Schistosoma haematobium *in Zimbabwean populations living in high and low schistosome infection areas. Derp1- and schistosome-specific IgE and IgG4 antibodies were quantified by ELISA before and 6 weeks after anti-helminthic treatment. Following treatment, there were changes in the immune responses, which varied with place of residence. After allowing for the effects of sex, age and baseline infection intensity, there was no significant treatment effect on the change in anti-schistosome IgE and IgG4 in the high infection area. However, the anti-schistosome IgE/IgG4 ratio increased significantly, while anti-Derp1 IgE responses decreased as a result of treatment. In the low infection area, treatment resulted in a significant increase in anti-worm IgE levels, but there was no significant treatment effect on anti-schistosome or anti-Derp1 IgE/IgG4 ratios. Thus, the study shows that the level of schistosome endemicity affects the host responses to schistosome and mite antigens following anti-helminthic treatment.*

## INTRODUCTION

Schistosomiasis remains one of the most important tropical human infections in terms of parasite-induced morbidity and mortality (1). The disease burden accounts for up to 70 million disability-adjusted life years (DALYs) annually (1), and it continues to threaten people in developing countries because of poor sanitation and lack of safe water sources. On the other hand, some epidemiological and animal studies suggest protective effects of helminth parasites including schistosomes and gastrointestinal nematodes against allergy and autoimmune disorders (2–4). The exact mechanisms involved in the relationship between parasitic infections and allergy/autoimmune disorders are poorly understood (5–7). There are studies suggesting that parasitic infections are protective (2,8) as well as studies suggesting the opposite (9). Possible explanations of these contrasting findings include differences in the intensity of the parasite infection, the parasite species and host genetic factors (3,5). Nonetheless, the relationship between helminth infections and allergy is believed to have an immunological basis. The general consensus is that exposure to either helminth parasites or allergens induces effector T-helper cell type 2 (Th2)-mediated immunity characterized by high IgE titres. However, in the case of helminth infection, these immune responses are modulated and there is evidence, at least in schistosome infection, to suggest that Th2 cells are impaired, with reduced responsiveness with chronicity of infection [rev. by Maizels *et al.* (10)].

The current method of control for schistosome infection is the treatment of infected people with the anti-helminthic drug praziquantel (PZQ). Although there have been several detailed, albeit conflicting, studies on the effects of anti-helminthic treatment for intestinal helminths on atopy (9,11–13), there are no extensive studies on the effects of the anti-helminthic PZQ in *Schistosoma haematobium* infections. In the light of the fact that the different life histories and biologies of helminths (e.g. nematodes vs. trematodes) result in different host-immunomodulatory dynamics (14), it is important to determine the effects of PZQ treatment for *S. haematobium* infections in endemic areas. Previous studies have shown that treatment for schistosome infections alters schistosome-specific immune responses (15–18). Furthermore, we have recently demonstrated that atopic responses are inversely associated with schistosome infection levels (19). However, it is currently unknown how different pre-treatment schistosome infection levels impact on atopic reactivity following anti-helminthic treatment. Thus, the aim of this study was to determine whether different pre-treatment schistosome infection levels and transmission dynamics altered the effects of PZQ treatment on allergen-specific antibody responses. To investigate this, the study was conducted in two villages with differing schistosome infection levels.

Levels of IgE and IgG4 against schistosome adult worm and egg antigens as well as those against the house dust mite *Dermatophagoides pteronyssinus* (Derp1) allergen – one of the most important allergen in clinical allergy (20) and prevalent in Zimbabwe (21) – were quantified before a single dose of PZQ was given and 6 weeks later. The aim was to investigate the dynamics of the relationship between atopic responses and schistosome-specific responses when pre-existing schistosome infection is cleared and newly acquired infection (if any) not yet patent (22) in human populations. The study focused on IgE and IgG4 antibody responses directed against schistosomes and the house dust mite because high levels of parasite-specific IgE are associated with resistance to infection/re-infection while parasite-specific IgG4 is believed to be a modulator of IgE effector responses (18,23,24). These antibodies are also important in clinical allergy where allergen-specific IgE antibodies are indicative of an allergic phenotype (25), while IgG4 antibodies are associated with improvement in allergic symptoms following immunotherapy or natural recovery (26–28). The relative proportions of these antibodies (or the balance between them) are therefore a key feature in humoral immunity against schistosomes (29–31) or predictors of clinical manifestations of atopy (28,32). We have already demonstrated in a previous study that atopy is slightly more prevalent in people resident in the low schistosome infection area compared to the high infection area (19). Furthermore, we reported that the levels of atopic responses were negatively associated with schistosome infection intensity. Thus, we hypothesize that the effect of treatment on the levels of schistosome-specific and allergen-specific IgE and IgG4 responses will vary between the villages of different levels of schistosome infection.

## MATERIALS AND METHODS

### Study design

The study was comparative, contrasting the effects of PZQ treatment on the levels of atopic responses as well as schistosome-specific antibody responses in high vs. low schistosome infection villages. Differences in infection levels reflect differences in infection transmission rates and history of infection (33). Subjects in the high infection village accumulate infection more rapidly, acquiring higher infection intensities at a younger age than their counterparts in the low infection village (33).

The two villages included in this study are classified as a high infection area (schistosome prevalence > 50%) and a low infection area (schistosome prevalence < 10%) based on the World Health Organization’s guidelines for areas endemic for *S. haematobium* infection (34). WHO recommends PZQ treatment schedules based on these transmission categories. Thus, the comparison made in this study is a representation of the field setting for the different levels of schistosome endemicity, allowing the comparison of the effects of PZQ treatment in these different populations.

### Study area and population

The study was conducted in two villages, Magaya and Chitate, in the Mashonaland East Province of Zimbabwe where *S. haematobium* is endemic. In this area, as in most rural regions in Zimbabwe (35,36), the prevalence of soil-transmitted helminths and *Schistosoma mansoni* is low, while *S. haematobium* is the most prevalent helminth infection. In addition, this study area was classified under the sporadic transmission regions with low *Plasmodium* transmission and malaria by a revised stratification based on national parasite prevalence surveys (37,38), Health Management Information Systems (HMIS) data, entomological data and expert opinion.

The study villages are in close proximity within a 10 km range of each other, and villagers are of similar ethnicity (Shona) and socioeconomic background (rural subsistence farmers). Safe water and sanitation coverage are equally poor in the villages (as assessed by questionnaire). The only difference between the villages is the seasonality of the rivers that provide habitats for schistosome intermediate host snails. Magaya village is characterized by perennial rivers that lead to high transmission rates of schistosome parasites compared to Chitate village, which is characterized by seasonal streams. In addition, households in Magaya are built along rivers, whereas in Chitate, they are more dispersed and built further from the rivers (surveyed by GPS mapping). Human contact with water potentially harbouring cercariae, the infective stage of schistosomes, is frequent (assessed by questionnaire) in this area because of insufficient safe water and sanitation facilities.

### Ethical statement

Permission to conduct the study in the region was obtained from the Provincial Medical Director, while institutional and ethical approvals were received from the University of Zimbabwe and the Medical Research Council of Zimbabwe, respectively. All participants had the aims and procedures of the project explained fully in the local language, Shona, and written consent was obtained before enrolment into the study. For young children, written consent was obtained from parents/guardians. After collection of all samples, all compliant participants were offered anti-helminthic treatment with the recommended dose of PZQ (40 mg/kg of body weight).

### Sample collection

Stool and urine samples were collected on three consecutive days for the diagnosis of schistosome and geo-helminth infections. *S. mansoni* and geo-helminths were diagnosed by stool egg count via the Kato Katz technique (39), while *S. haematobium* was diagnosed and quantified via urine filtration followed by microscopic egg count (40). Infection intensity was calculated as the mean egg count obtained from two or three specimens. Venous blood was collected, from each participant, into silicone-coated tubes without anticoagulant and was used to obtain sera for antibody assays. Malaria status was determined by blood smears and confirmed by a serological Paracheck dipstick (Orchid Biomedical systems, Goa, India).

### Inclusion criteria

**Figure 1:**
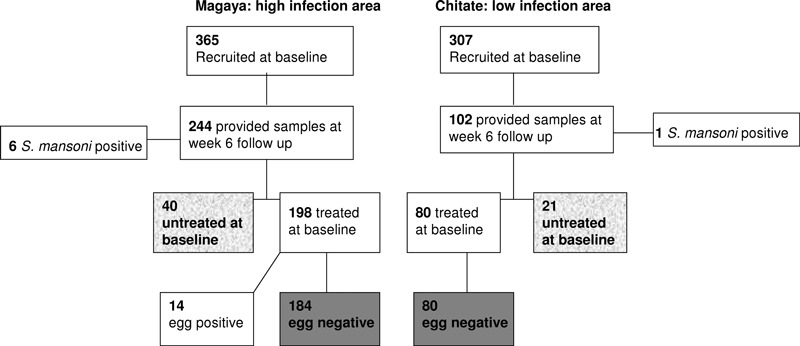
Flow diagram showing numbers of recruited and followed up participants. The highlighted boxes represent people included in the present analysis (treated in plain grey and untreated in grey texture).

To be included in the study, participants had: (i) to be lifelong residents of the village (assessed by questionnaire); (ii) to not previously have received anti-helminthic treatment; (iii) to provide, at baseline and 6 weeks post-treatment, urine and stool samples for helminth diagnosis and blood samples for serological assays; (iv) to be negative for geo-helminth or *S. mansoni* infections; and (v) to be schistosome egg negative 6 weeks after anti-helminthic treatment if they were treated with PZQ. A total of 325 individuals fulfilled the inclusion criteria (Figure 1) and formed the study populations. 61 individuals who refused treatment (40 from the high infection areas and 21 from the low infection area) for religious reasons but wished to remain in the study effectively formed the untreated control groups. Characteristics of the study populations are detailed in Table 1.

**Table 1:** Description of the study population

	High infection area		Low infection area	
		
	Treated	Untreated	Treated	Untreated
Total, *N*	184	40	80	21
Age groups				
Age group 1 (6–10 years)	105	30	42	16
Age group 2 (11–15 years)	60	5	22	2
Age group 3 (16+)	19	5	16	3
Sex ratio (M : F)	0·8	1·7	0·6	0·9
Mean age (range)	12·8 years (6–84)	12 years (6–62)	17 years (6–86)	13·3 years (6–55)
Mean infection intensity at baseline (range)	26·2 (0–502·3) GM: 0·62	5·9 (0–50·7) GM: 0·28	0·43 (0–20) GM: 0·05	0·8 (0–8·7) GM: 0·11
Egg positive cases at baseline (prevalence)	97 (52·7%)	10 (25%)	5 (6·25%)	3 (14·3%)

Infection levels [mean egg count/10 mL urine and geometric mean (GM)], sex ratio (M: male and F: female) and age range for treated and untreated individuals selected from the high and low infections areas

### Antigens

Lyophilized soluble *S. haematobium* adult worms (males and females at a ratio of 1 : 1) (SWAP) and egg antigens (SEA) were obtained from the Theodor Bilharz Institute (Giza, Egypt) and reconstituted as recommended by the manufacturer. The parasite strain is one used for previous immuno-epidemiological studies in Zimbabwe (15,16). Natural Derp1 allergen (NA-DP-1-2, purified by affinity chromatography) was obtained from Indoor Biotechnologies Ltd (Charlottesville, VA, USA).

### Antibody assays

A standard indirect enzyme-linked immunosorbent assay (ELISA) (33) was optimized and used to quantify the levels of antibodies (IgE and IgG4) directed against *S. haematobium* adult worm antigen (SWAP), SEA and Derp1 allergen. ELISAs for pre- and post-treatment samples were conducted in the same period of time and by the same person to minimize experimental variations. Serum samples for pre- and post-treatment were not run on the same plate but six individual sera were run on each ELISA plate and these were statistically comparable. Furthermore, a pool of responders (positive controls) was run on each plate and readings were comparable across plates, that is, the % CV was <20% for all comparisons and readings were below the critical values (see 41); therefore, no correction factor was used.

#### ELISAs for schistosome antigens

Microtitre plates were coated overnight at 4°C with 100 μL/well of antigen at 5 μg/mL for SWAP and 10 μg/mL for SEA in carbonate bicarbonate buffer (pH = 9·6) and then washed once with PBS 0·03% Tween-20 (which was used for all subsequent washes). Plates were blocked with 5% skimmed milk in PBS/0·05% Tween-20 for 2 h before 100 μL of the serum samples were added diluted at 1 : 20 for IgE, 1 : 100 for IgG4 anti-SWAP and 1 : 400 for IgG4 anti-SEA. Plates were incubated for 2 h at a temperature of 37°C. After washing three times, 100 μL of anti-human horseradish peroxide-conjugated IgE (Sigma, London, UK) or IgG4 (The Binding Site Birmingham, UK) diluted at 1 : 1000 except for IgG4 anti-SWAP (1 : 500) and IgE anti-SWAP (1 : 250). After 1-h incubation at 37°C followed by six washes, 100 μL of the substrate (ABTS; Southern Biotech, Birmingham, AL, USA) was added. The reaction was stopped after 15-min incubation at room temperature for IgG4 and 30 min at 37°C for IgE, using 25% HCL; absorbance readings of all wells were performed at 405 nm. All samples were assayed in duplicate, and a blank control containing no sera was included (also in duplicate) on each plate and the background absorbance of reagents in the absence of serum was subtracted from all readings.

#### ELISA for Derp1 allergen

The protocol above described was used with modifications: plates were coated with 50 μL/well of Derp1 at 5 μg/mL and samples diluted 1 : 10. Detection antibodies (IgE from Sigma and IgG4 from The Binding Site) were diluted 1 : 1000, and the reaction time for the substrate was 30 min at 37°C.

### Statistical analysis

In this study, parametric tests were performed rather than non-parametric tests as these are more powerful and allow studying the relationship between epidemiological variables while controlling for confounding factors. However, the raw data were transformed to fulfil the assumptions of parametric tests [normal distribution of residuals, homogeneity of variance, linearity and orthogonality (42)]. Thus, antibody data were square root-transformed while infection intensity was log_10_(*x* + 1)-transformed and these gave normally distributed residual plots (of the otherwise skewed raw data). Age was categorized into three age groups reflecting epidemiological patterns of infection to allow the non-linear relationship between age and infection to be fitted in the models used (42). These age groups and the sample sizes for each group in each village and treatment status are illustrated in Table 1. Schistosome infection prevalence and intensity at baseline were compared between the two villages by Pearson chi-squared test and analysis of variance (anova), respectively. To test whether there were significant changes in antibody levels between the two time points (baseline and 6 weeks post-treatment), and whether these were owing to treatment or other variables (age, sex, village, baseline infection), a repeated measures general linear model (GLM) was conducted. In this model, antibody levels (square root-transformed) were the dependent variables (two levels, pre- and post-treatment), while the independent variables were age (categorical, group 1, 6–10 years; group 2, 11–15 and group 3, 16+), sex (categorical, male/female), village (categorical, HIA/LIA), infection intensity at baseline (covariate, log_10_*x* + 1-transformed) and treatment status (categorical, treated/untreated). Sequential sum of squares (type I) was used in this model to allow the hierarchical decomposition of the sources of variation in the dependent variable because some independent variables share information [non-orthogonality, see (42) for review]. The variable ‘treatment status’ was entered last in this model to allow for the effects of all other independent variables before testing for the effects of treatment. As this initial analysis showed that the changes in some antibody levels differed between the villages of residence, the data were partitioned by village to test the hypothesis that the effect of treatment on the magnitude of antibody change is dependent on cumulative history of infection. Thus, the effect of treatment on the magnitude of antibody and ratios changes (determined as post-treatment values minus pre-treatment values) was determined by the analysis of variance in each village, allowing for the variations owing to host age, sex and baseline infection intensity. In this analysis, the sequential sums of squares were also used to calculate the test statistics with treatment status entered last in the model. To test the hypothesis that baseline infection intensity affects the magnitude of change in antibody levels, the variable ‘infection intensity’ was entered after ‘sex’ and ‘age’ in this model.

All statistical analyses were conducted in pasw (IBM corporation, Armonk, NY, USA) statistics 17, and *P*-values <0·05 were considered significant.

## Results

### Baseline infection intensity and prevalence differ significantly between the two villages

**Figure 2:**
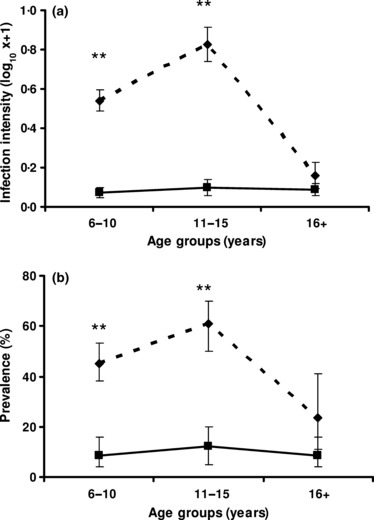
Prevalence and infection intensity (egg count per 10ml) by age in the study area. Overall infection intensity (a) and prevalence (b) for the population from which the study cohorts were chosen. Infection levels (log-transformed for infection intensity and percentage of positive cases for prevalence) are plotted against age groups in the high infection area (doted line) and the low infection area (plain line). Bars represent standard error of the means for infection intensity and 95% CI for the prevalence. Asterisks represent significant differences at *P* < 0·01 in each group between the two villages.

At baseline, schistosome infection levels were significantly higher in Magaya than Chitate with a prevalence of 47·5% vs. 9·3% (χ² = 100·5, d.f. = 1, *P* < 0·001) and mean infection intensity of 26·2 eggs per 10 mL vs. 0·9 eggs per 10 mL (*F*_1, 580_ = 79·748, *P* < 0·001), respectively. When partitioned by age groups, there were significant differences in infection levels in the two areas in younger age groups (Figure 2). There was an earlier peak of infection in Magaya than Chitate, although the peak in Chitate was not statistically significant, and a significant age and village interaction (*F*_1, 579_ = 6·963, *P* = 0·009).

### Overall change in antibody levels between the two time points

Results from the repeated measures GLM analysis between pre- and post-treatment antibody levels (Table 2) show significant changes in anti-schistosome and anti-Derp1 antibody responses over time (i.e. time between baseline and 6 weeks post-treatment). Although there were changes between the two time points in most schistosome antibody levels as well as in anti-allergen responses, only changes in anti-SWAP IgE and Derp1 IgE were related to treatment as shown by the significant time*PZQ treatment interaction in Table 2. As a result of the significant change in anti-SWAP IgE, the ratio IgE/IgG4 also increased significantly in treated people compared to untreated people. However, this analysis showed that the temporal changes differed between the two villages (significant interaction time*village). Thus, for SWAP IgE, the increase was greater in the high transmission village; for SEA-IgE and the ratio IgE/IgG4 SEA, there was a slight increase in the high transmission village and a decrease in the low transmission village; for Derp1 IgE and the ratio IgE/IgG4 –Derp1, the decline was greater in the high transmission village; and for Derp1 IgG4, the increase was greater in the low transmission village. A further analysis was conducted (as detailed below) to determine the effect of treatment in each village.

**Table 2:** Results from a repeated measures general linear model (GLM) analysis of the variations in antibody levels

Dependent pair (pre- and post-treatment)	Time *F* (*P*)	Time^*^sex *F* (*P*)	Time^*^age *F* (*P*)	Time^*^village *F* (*P*)	Time^*^infection intensity *F* (*P*)	Time^*^PZQ treatment *F* (*P*)
Anti-schistosome responses						
SWAP IgE	**77·178 (<0·001)**	0·040 (0·842) f<m	0·108 (0·898)	**13·925 (<0·001)H > L**	**19·561 (<0·001)**	**5·558 (0·019)T > U**
SWAP IgG4	**49·485 (<0·001)**	2·077 (0·15) f < m	**11·024 (<0·001)**	0·354 (0·552)H > L	**26·952 (<0·001)**	0·209 (0·648)T > U
SWAP IgE/IgG4	**4·72 (0·031)**	0·558 (0·455)f > m	1·778 (0·171)	0·095 (0·758)H > L	1·052 (0·306)	**4·342 (0·038)T > U**
SEA-IgE	**5·00 (0·026)**	1·315 (0·252)f < m	**5·828 (0·003)**	**50·299 (<0·001)H > L**	**15·534 (<0·001)**	3·193 (0·075)T < U
SEA-IgG4	0·323 (0·57)	0·279 (0·597)f < m	0·321 (0·726)	2·638 (0·105)H > L	**6·086 (0·014)**	2·368 (0·125)T > U
SEA-IgE/IgG4	2·070 (0·151)	0·44 (0·508) f > m	1·378 (0·254)	**36·535 (<0·001)H < L**	1·586 (0·209)	1·041 (0·308)T < U
Anti-allergen responses						
Derp1-IgE	**47·238 (<0·001)**	**6·281 (0·013)f > m**	**6·939 (0·001)**	**6·382 (0·012)H < L**	0·009 (0·926)	**4·902 (0·028)T > U**
Derp1-IgG4	**42·278 (<0·001)**	3·548 (0·061)f < m	**13·72 (<0·001)**	**34·706 (<0·001)H < L**	1·783 (0·183)	0·768 (0·382)T < U
Derp1-IgE/IgG4	**34·963 (<0·001)**	0·271 (0·603)f > m	**4·347 (0·014)**	**15·063 (<0·001)H > L**	3·786 (0·053)	3·414 (0·066)T < U

*F* (and *P*) values from repeated measures GLM analysis of the parameters explaining the variations in antibody responses (and ratios) over time (time here stands for the 6 weeks between pre- and post-treatment collection day). Significant *P*-values <0·05 are highlighted in bold. SWAP, soluble worm antigen preparation; SEA, soluble egg antigen; Derp1, *Dermatophagoides pteronyssinus* allergen 1; PZQ, praziquantel; f, female; m, male; H, high infection area; L, low infection area; T, treated; U: untreated.

### Differences in antibody responses between treated and untreated people from different villages

**Figure 3:**
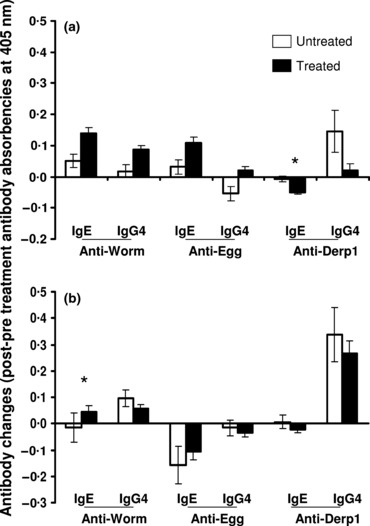
Comparison of changes in antibody levels. Mean antibody changes for the study populations are shown for treated (black columns) and untreated (white columns) people in the high infection area (a) and the low infection area (b) with standard error of means. Antibody changes are calculated as the difference between post-treatment square root-transformed levels and pre-treatment square root-transformed levels. Asterisks represent significant differences between treated and untreated people at *P* < 0·05, which are obtained from the analysis of variance after allowing for the variation owing to the potential confounding variables – host age, sex and pre-treatment infection intensity.

**Figure 4:**
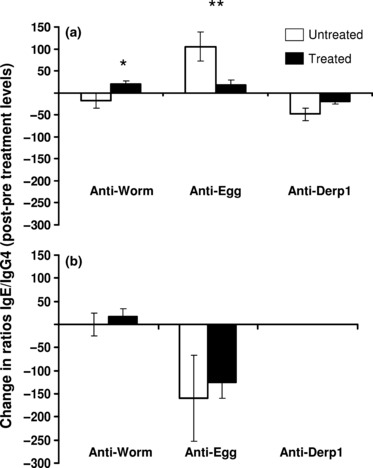
Comparison of changes in the ratios. Mean changes in ratios IgE/IgG4 for treated (black columns) and untreated (white columns) people are shown for the high infection area (a) and the low infection area (b) with standard error of means. Asterisks represent significant differences between treated and untreated people at *P < 0·05 and **P < 0·01 obtained from the analysis of variance after allowing for the variation owing to the potential confounding variables – host age, sex and pre-treatment infection intensity.

The data were partitioned by residential village and the change in antibody levels analysed by anova after allowing for the effects of host age, sex and baseline infection intensity. This analysis (Table 3 and Figures 3 and 4) showed that in the high infection area, although no significant difference occurred in the individual antibodies (Figure 3), the ratio anti-SWAP IgE/IgG4 rose significantly while that of anti-SEA was significantly lower in treated vs. untreated people (Figure 4). Derp1-specific IgE levels declined significantly in treated people compared to untreated people in the high infection area. In the low infection area, only the change (increase) in SWAP IgE was significantly different between treated and untreated people and there was no significant difference in the levels of allergen-specific responses in treated vs. untreated people (Figure 3).

**Table 3:** Results from a multivariate general linear model (GLM) analysis of the effects of treatment, by village, on the difference between pre- and post-treatment antibody levels

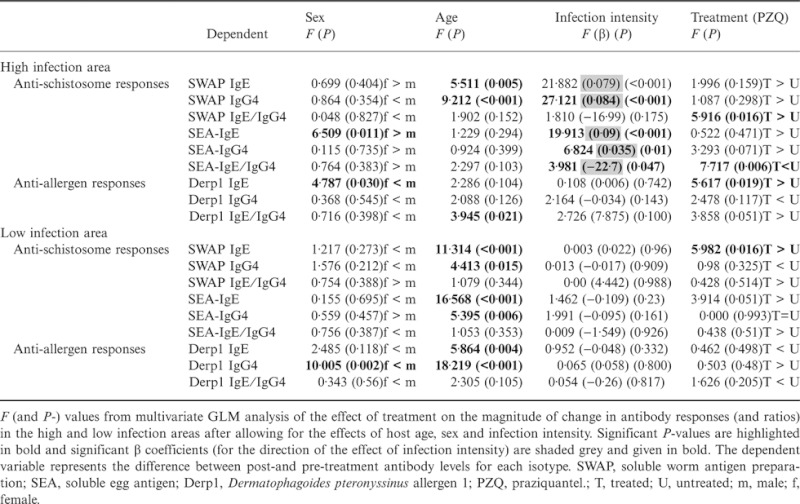

Baseline infection intensity was significantly associated with the treatment-induced antibody change for anti-schistosome responses but not anti-Derp1 responses in the high infection area. As shown in Table 3, there were significant effects of infection intensity on IgE and IgG4 against the adult worm and egg antigens (positive β coefficients: positive association) as well as the ratio IgE/IgG4 against the egg antigens (negative β coefficient: negative association) in the high infection area.

## Discussion

With the increasing calls for concerted helminth control efforts to meet Millennium Development Goal 6, it is important that the consequences of anti-helminthic treatment are fully understood. On the one hand, there is mounting evidence that anti-helminthic treatment not only transiently reduces infection, but also has longer term benefits in terms of morbidity control and the development of parasite-specific immune responses associated with resistance to re-infection (18,43–45). On the other hand, there are several studies suggesting that anti-helminthic treatment increases the incidence of atopic reactivity (11,12), although there are some studies showing the opposite effect (9). Heterogeneities in parasite transmission dynamics may influence the effect of anti-helminthic treatment on atopic reactivity as well as parasite-specific responses. Thus, we investigated the effect of PZQ treatment on anti-mite as well as anti-schistosome IgE and IgG4 antibody responses in two villages of differing schistosome infection levels. The two villages selected represented two categories of schistosome endemicity defined by the WHO (34), that is, an area of low infection levels (prevalence <10% as occurs in Chitate) and an area of high infection (prevalence >50% as occurs in Magaya), which guide anti-helminthic treatment schedules. In a previous study, we demonstrated that there was a difference in the levels of atopic responses, which was related to current levels of schistosome infection (19). We also demonstrated significant differences in the levels of schistosome-specific and Derp1-specific IgE and IgG4 responses.

This current study evaluated the effects of anti-helminthic treatment in these villages because the WHO guidelines recommend treatment in villages with both these levels of schistosome endemicity. Our study shows that 6 weeks after anti-helminthic treatment, there were differences in schistosome and Derp1-specific antibody changes in the two villages. The enrolment of untreated controls in this group enables the distinction to be made between treatment-related changes in the immune responses and temporal differences occurring owing to the natural development of immune responses (especially in infected untreated people) and potential stochastic variations. Indeed, there were temporal differences in the levels of immune responses, but only differences in treated people compared to untreated people could be attributed to PZQ treatment.

Treatment was associated with a significant increase in the ratio IgE/IgG4 against schistosome adult worm antigens in the high infection area. High levels of parasite-specific IgE have been associated with resistance to schistosome infection while IgG4 are associated with susceptibility (18,23). The increase in the levels of anti-SWAP IgE over IgG4 (ratio IgE/IgG4) following chemotherapy in this study supports a role of PZQ in the development of putatively protective acquired immunity as it has been reported previously (18,23). In the low infection area, these changes are less apparent possibly due to low levels of infection (and therefore low levels of parasite-specific antibodies) (46), but levels of anti-worm IgE antibodies increased significantly in treated individuals in this area. This is consistent with the result showing a positive association between infection intensity and the magnitude of change in antibody levels (represented by the β value from the anova) in the high infection area, which is not apparent in the low infection area, where infection levels may be too low for a distinct association to be detectable.

Variations in anti-schistosome egg antibody responses following PZQ treatment have been reported (43) and studies on *Schistosoma japonicum* associated anti-egg IgE responses with resistance to re-infection (24). In the present study, the magnitude of increase in the IgE/IgG4 ratio against SEA in the high infection area was lower in treated than untreated people, reflecting an increase in both SEA-IgE and SEA-IgG4 following treatment. Furthermore, this ratio was negatively associated with pre-treatment infection intensity, suggesting that, in contrast to anti-worm responses, SEA-IgG4 increased more than SEA-IgE in people heavily infected prior treatment. This is suggestive of, although not conclusive evidence for, the immunomodulatory role of IgG4 (30) following the rapid release of parasite ‘inflammatory’ egg antigens after PZQ treatment.

Similar to schistosome-specific responses, there were temporal differences in the allergen-specific responses. However, only changes in anti-Derp1 IgE levels in the high infection area differed in treated vs. untreated people. In the treated people, anti-Derp1 IgE levels declined significantly compared to untreated people. However, there were no significant changes in the ratio of the anti-Derp1 IgE/IgG4, possibly because of the increase in anti-DerpIgG4. The reason for this increase, which occurred in individuals with low or no infection at baseline (treated and untreated), requires further investigations [e.g. seasonal variations (47)].

IgE mediates the pathological manifestations of allergy (25,26), and we have shown that these antibodies correlate with skin sensitization in this population (19). Thus, a decline in the levels of this antibody may result in reduced clinical atopy.

The decline in IgE anti-Derp1 observed following treatment in the present study is consistent with findings in Gabon (12). These authors reported a significant increase in skin sensitization, while allergen-specific IgE slightly declined, over the course of a 30 months of PZQ –mebendazole treatment for helminth-infected children.

Because atopy is inversely associated with schistosome infection (2,19,48), this decline in allergen-specific IgE may reflect the mechanisms involved in PZQ -associated desensitization (49). These authors demonstrated that blood basophils from *S. mansoni*-infected individuals were desensitized to schistosome egg and worm antigens after treatment. Sensitized basophils release histamine and cytokines, including IL-4 capable of inducing Th2-IgE responses (50,51). Furthermore, it has been shown that serum IgE levels are closely related to FcεRI expression on basophils (52,53). Therefore, basophil desensitization may have impacted on the levels of Derp1 IgE reported in the current study.

However, because anti-schistosome IgE increased, it is also possible that the polyclonal stimuli of ‘cross-reactive’ IgE, characteristic of helminth infections (54–56), has been removed with treatment and a more specific anti-parasite response produced. This is in line with findings that anti-schistosome chemotherapy induces qualitative changes in serological recognition of parasite antigen (16) and increases cellular reactivity to parasite antigens (17,57,58).

The lack of changes in the allergen-specific responses in the low infection area is not surprising because the relationship between atopic responses and schistosome infection was weak due to low levels of infection intensity (19). In the previous study, we demonstrated that it was heavy infections that were associated with lower levels of atopy. Thus, treating this population with low levels of schistosome infections would have less impact on both the parasite-specific responses and atopic responses.

Several studies have been conducted in different populations investigating the effects of anti-helminthic treatment on atopy. These studies have reported different effects from having no effect on (13), worsening (12) and improving (9) atopy. Potential explanations for these differences include different parasite phyla (nematodes vs. trematodes), infection intensity (low vs. high) and environmental factors (59–62). Thus, meta-analyses approach and comparative studies such us our present studies are powerful in addressing the effects of heterogeneities in this complex association between atopy and helminth infection.

Taken together, these findings show that within 6 weeks, a single dose of PZQ treatment can affect schistosome-specific and allergen-specific IgE and IgG4 responses. However, the effects of PZQ treatment on these responses depend on schistosome endemicity levels. Thus, in a high infection area, PZQ treatment can increase antibody levels associated with resistance to schistosome infection/re-infection while decreasing the levels of sensitization to the house dust mite. With the current WHO recommendations to implement control programs in all endemic areas for schistosomiasis, this study highlights the need to investigate the effect of treatment at different levels of endemicity to predict long-term consequences of deworming. There was no evidence from this study to suggest that chemotherapeutic treatment for schistosomiasis may exacerbate atopic responses in endemic areas. The study does not preclude the possibility that deworming may impact on allergic diseases and further longer term longitudinal comparative studies are required.

## References

[1] Hotez PJ, Fenwick A (2009). Schistosomiasis in Africa: an emerging tragedy in our new global health decade. PLoS Negl Trop Dis.

[2] van den Biggelaar AH, van Ree R, Rodrigues LC (2000). Decreased atopy in children infected with *Schistosoma haematobium*: a role for parasite-induced interleukin-10. Lancet.

[3] Smits HH, Hammad H, van Nimwegen M (2007). Protective effect of *Schistosoma mansoni* infection on allergic airway inflammation depends on the intensity and chronicity of infection. J Allergy Clin Immunol.

[4] Mutapi F, Imai N, Nausch N (2011). Schistosome infection intensity is inversely related to auto-reactive antibody levels. PLoS One.

[5] Cooper PJ (2009). Interactions between helminth parasites and allergy. Curr Opin Allergy Clin Immunol.

[6] Osada Y, Kanazawa T (2010). Parasitic helminths: new weapons against immunological disorders. J Biomed Biotechnol.

[7] Feary J, Britton J, Leonardi-Bee J (2011). Atopy and current intestinal parasite infection: a systematic review and meta-analysis. Allergy.

[8] Supali T, Djuardi Y, Wibowo H, van Ree R, Yazdanbakhsh M, Sartono E (2010). Relationship between different species of helminths and atopy: a study in a population living in helminth-endemic area in Sulawesi, Indonesia. Int Arch Allergy Immunol.

[9] Lynch NR, Palenque M, Hagel I, DiPrisco MC (1997). Clinical improvement of asthma after anthelminthic treatment in a tropical situation. Am J Respir Crit Care Med.

[10] Maizels RM, Pearce EJ, Artis D, Yazdanbakhsh M, Wynn TA (2009). Regulation of pathogenesis and immunity in helminth infections. J Exp Med.

[11] Lynch NR, Hagel I, Perez M, Di Prisco MC, Lopez R, Alvarez N (1993). Effect of anthelmintic treatment on the allergic reactivity of children in a tropical slum. J Allergy Clin Immunol.

[12] van den Biggelaar AH, Rodrigues LC, van Ree R (2004). Long-term treatment of intestinal helminths increases mite skin-test reactivity in Gabonese schoolchildren. J Infect Dis.

[13] Cooper PJ, Chico ME, Vaca MG (2006). Effect of albendazole treatments on the prevalence of atopy in children living in communities endemic for geohelminth parasites: a cluster-randomised trial. Lancet.

[14] Bourke CD, Maizels RM, Mutapi F (2011). Acquired immune heterogeneity and its sources in human helminth infection. Parasitology.

[15] Mutapi F, Ndhlovu PD, Hagan P (1998). Chemotherapy accelerates the development of acquired immune responses to *Schistosoma haematobium* infection. J Infect Dis.

[16] Mutapi F, Burchmore R, Mduluza T (2005). Praziquantel treatment of individuals exposed to *Schistosoma haematobium* enhances serological recognition of defined parasite antigens. J Infect Dis.

[17] Grogan JL, Kremsner PG, Deelder AM, Yazdanbakhsh M (1996). Elevated proliferation and interleukin-4 release from CD4 + cells after chemotherapy in human *Schistosoma haematobium* infection. Eur J Immunol.

[18] Black CL, Muok EM, Mwinzi PN (2010). Increases in levels of schistosome-specific immunoglobulin E and CD23(+) B cells in a cohort of Kenyan children undergoing repeated treatment and reinfection with *Schistosoma mansoni*. J Infect Dis.

[19] Rujeni N, Nausch N, Bourke CD (2012). Atopy is inversely related to schistosome infection intensity: a comparative study in Zimbabwean villages with distinct levels of *Schistosoma haematobium* infection. Int Arch Allergy Immunol.

[20] Taketomi EA, Silva DA, Sopelete MC, Gervasio AM, Alves R, Sung SJ (2006). Differential IgE reactivity to Derp1 and Derp2 allergens of Dermatophagoides pteronyssinus in mite-sensitized patients. J Investig Allergol Clin Immunol.

[21] Westritschnig K, Sibanda E, Thomas W (2003). Analysis of the sensitization profile towards allergens in central Africa. Clin Exp Allergy.

[22] Tchuente LA, Shaw DJ, Polla L, Cioli D, Vercruysse J (2004). Efficacy of praziquantel against *Schistosoma haematobium* infection in children. Am J Trop Med Hyg.

[23] Hagan P, Blumenthal UJ, Dunn D, Simpson AJ, Wilkins HA (1991). Human IgE, IgG4 and resistance to reinfection with *Schistosoma haematobium*. Nature.

[24] Zhang Z, Wu H, Chen S (1997). Association between IgE antibody against soluble egg antigen and resistance to reinfection with Schistosoma japonicum. Trans R Soc Trop Med Hyg.

[25] Johansson SG, Bieber T, Dahl R (2004). Revised nomenclature for allergy for global use: report of the Nomenclature Review Committee of the World Allergy Organization, October 2003. J Allergy Clin Immunol.

[26] Bullock RJ, Barnett D, Howden ME (2005). Immunologic and clinical responses to parenteral immunotherapy in peanut anaphylaxis--a study using IgE and IgG4 immunoblot monitoring. Allergol Immunopathol (Madr).

[27] Tseng SH, Fu LS, Nong BR, Weng JD, Shyur SD (2008). Changes in serum specific IgG4 and IgG4/IgE ratio in mite-sensitized Taiwanese children with allergic rhinitis receiving short-term sublingual-swallow immunotherapy: a multicenter, randomized, placebo-controlled trial. Asian Pac J Allergy Immunol.

[28] Savilahti EM, Rantanen V, Lin JS (2010). Early recovery from cow’s milk allergy is associated with decreasing IgE and increasing IgG4 binding to cow’s milk epitopes. J Allergy Clin Immunol.

[29] Vereecken K, Naus CW, Polman K (2007). Associations between specific antibody responses and resistance to reinfection in a Senegalese population recently exposed to *Schistosoma mansoni*. Trop Med Int Health.

[30] Maizels RM, Yazdanbakhsh M (2003). Immune regulation by helminth parasites: cellular and molecular mechanisms. Nat Rev Immunol.

[31] Jiz M, Friedman JF, Leenstra T (2009). Immunoglobulin E (IgE) responses to paramyosin predict resistance to reinfection with Schistosoma japonicum and are attenuated by IgG4. Infect Immun.

[32] Palomares O, Yaman G, Azkur AK, Akkoc T, Akdis M, Akdis CA (2010). Role of Treg in immune regulation of allergic diseases. Eur J Immunol.

[33] Mutapi F, Ndhlovu PD, Hagan P, Woolhouse ME (1997). A comparison of humoral responses to *Schistosoma haematobium* in areas with low and high levels of infection. Parasite Immunol.

[34] World Health Organization (WHO) (2002). Prevention and Control of Schistosomiasis and Soil Transmitted Helminthiasis, Report of a WHO Expert Committee.

[35] Midzi N, Sangweme D, Zinyowera S (2008). The burden of polyparasitism among primary schoolchildren in rural and farming areas in Zimbabwe. Trans R Soc Trop Med Hyg.

[36] Taylor P, Makura O (1985). Prevalence and distribution of schistosomiasis in Zimbabwe. Ann Trop Med Parasitol.

[37] Mabaso ML, Craig M, Vounatsou P, Smith T (2005). Towards empirical description of malaria seasonality in southern Africa: the example of Zimbabwe. Trop Med Int Health.

[38] Mabaso ML, Vounatsou P, Midzi S, Da Silva J, Smith T (2006). Spatio-temporal analysis of the role of climate in inter-annual variation of malaria incidence in Zimbabwe. Int J Health Geogr.

[39] Katz N, Chaves A, Pellegrino J (1972). A simple device for quantitative stool thick-smear technique in *Schistosomiasis mansoni*. Rev Inst Med Trop Sao Paulo.

[40] Mott KE (1983). A reusable polyamide filter for diagnosis of *S. haematobium* infection by urine filtration. Bull Soc Pathol Exot Filiales.

[41] Reed GF, Lynn F, Meade BD (2002). Use of coefficient of variation in assessing variability of quantitative assays. Clin Diagn Lab Immunol.

[42] Mutapi F, Roddam A (2002). p values for pathogens: statistical inference from infectious-disease data. Lancet Infect Dis.

[43] Mutapi F, Hagan P, Woolhouse ME, Mduluza T, Ndhlovu PD (2003). Chemotherapy-induced, age-related changes in antischistosome antibody responses. Parasite Immunol.

[44] Black CL, Mwinzi PN, Muok EM (2010). Influence of exposure history on the immunology and development of resistance to human *Schistosomiasis mansoni*. PLoS Negl Trop Dis.

[45] WHO Expert Committee on the Control of Schistosomiasis (1993). The Control of schistosomiasis: Second report of the WHO Expert Committee.

[46] Mutapi F, Burchmore R, Mduluza T, Midzi N, Turner CM, Maizels RM (2008). Age-related and infection intensity-related shifts in antibody recognition of defined protein antigens in a schistosome-exposed population. J Infect Dis.

[47] Nahm DH, Park HS, Kim CW, Park JW, Hong CS (1998). Seasonal variation of IgG subclass antibodies to house dust mite in sera from mite-sensitive asthmatic patients. Ann Allergy Asthma Immunol.

[48] Araujo MI, Lopes AA, Medeiros M (2000). Inverse association between skin response to aeroallergens and *Schistosoma mansoni* infection. Int Arch Allergy Immunol.

[49] Satti MZ, Cahen P, Skov PS (2004). Changes in IgE- and antigen-dependent histamine-release in peripheral blood of *Schistosoma mansoni*-infected Ugandan fishermen after treatment with praziquantel. BMC Immunol.

[50] Yoshimoto T, Yasuda K, Tanaka H (2009). Basophils contribute to T(H)2-IgE responses in vivo via IL-4 production and presentation of peptide-MHC class II complexes to CD4 + T cells. Nat Immunol.

[51] Min B, Prout M, Hu-Li J (2004). Basophils produce IL-4 and accumulate in tissues after infection with a Th2-inducing parasite. J Exp Med.

[52] Malveaux FJ, Conroy MC, Adkinson NF, Lichtenstein LM (1978). IgE receptors on human basophils. Relationship to serum IgE concentration. J Clin Invest.

[53] Saini SS, Klion AD, Holland SM, Hamilton RG, Bochner BS, Macglashan DW (2000). The relationship between serum IgE and surface levels of FcepsilonR on human leukocytes in various diseases: correlation of expression with FcepsilonRI on basophils but not on monocytes or eosinophils. J Allergy Clin Immunol.

[54] Lynch NR, Hagel I, Vargas M (1993). Effect of age and helminthic infection on IgE levels in slum children. J Investig Allergol Clin Immunol.

[55] Hagel I, Lynch NR, Perez M, Di Prisco MC, Lopez R, Rojas E (1993). Modulation of the allergic reactivity of slum children by helminthic infection. Parasite Immunol.

[56] Yazdanbakhsh M, Kremsner PG, van Ree R (2002). Allergy, parasites, and the hygiene hypothesis. Science.

[57] Colley DG, Barsoum IS, Dahawi HS, Gamil F, Habib M, el Alamy MA (1986). Immune responses and immunoregulation in relation to human schistosomiasis in Egypt. III. Immunity and longitudinal studies of in vitro responsiveness after treatment. Trans R Soc Trop Med Hyg.

[58] Joseph S, Jones FM, Walter K (2004). Increases in human T helper 2 cytokine responses to *Schistosoma mansoni* worm and worm-tegument antigens are induced by treatment with praziquantel. J Infect Dis.

[59] Prescott SL (2011). The influence of early environmental exposures on immune development and subsequent risk of allergic disease. Allergy.

[60] Flohr C, Quinnell RJ, Britton J (2009). Do helminth parasites protect against atopy and allergic disease?. Clin Exp Allergy.

[61] Smits HH, Everts B, Hartgers FC, Yazdanbakhsh M (2010). Chronic helminth infections protect against allergic diseases by active regulatory processes. Curr Allergy Asthma Rep.

[62] Cooper PJ, Barreto ML, Rodrigues LC (2006). Human allergy and geohelminth infections: a review of the literature and a proposed conceptual model to guide the investigation of possible causal associations. Br Med Bull.

